# Arthroscopic findings and accuracy of preoperative diagnosis in patients with chronic lateral ankle instability

**DOI:** 10.1002/jeo2.70739

**Published:** 2026-06-10

**Authors:** Pierre‐Henri Vermorel, Jordi Vega, Miki Dalmau‐Pastor, Andrea Pantalone, Javier Zaourak, Matteo Guelfi

**Affiliations:** ^1^ Department of Orthopaedic Surgery University Hospital Centre of Saint‐Étienne Saint‐Étienne France; ^2^ Laboratoire Interuniversitaire de Biologie de la Motricité Université Jean Monnet Saint‐Etienne, CHU Saint‐Etienne, Lyon 1, Université Savoie Mont‐Blanc Saint‐Etienne France; ^3^ Clinica Salus, Policlinico di Monza Alessandria Italy; ^4^ Foot and Ankle Unit iMove Traumatology Barcelona Spain; ^5^ MIFAS by Grecmip Merignac France; ^6^ Human Anatomy and Embryology Unit, Department of Pathology and Experimental Therapeutics, School of Medicine and Health Sciences University of Barcelona Barcelona Spain; ^7^ Department of Medicine and Aging Sciences University of Chieti‐Pescara Chieti Italy; ^8^ Foot and Ankle Unit CLIMBA Buenos Aires Argentina; ^9^ Casa di Cura Villa Montallegro Genoa Italy; ^10^ J Medical Turin Italy

**Keywords:** ankle arthroscopy, chronic ankle instability, diagnosis accuracy, intra‐articular lesions, microinstability

## Abstract

**Purpose:**

Management of chronic lateral ankle instability (CLAI) has significantly evolved in recent years, driven by an increased awareness of ankle microinstability and advances in minimally invasive techniques. The purpose of this study was to report arthroscopic findings in ankles with CLAI treated arthroscopically according to contemporary concepts and to evaluate the diagnostic accuracy of preoperative assessments. We hypothesized that current indications for arthroscopic treatment of CLAI increasingly involve milder instability, which are associated with a lower incidence of intra‐articular pathology than previously reported.

**Methods:**

A retrospective case series was conducted on 179 patients who underwent ankle arthroscopy for CLAI between 2020 and 2024. Ligamentous injuries were assessed both preoperatively (clinical examination and magnetic resonance imaging) and intra‐operatively (arthroscopy), including anterior talo‐fibular ligament's (ATFL) superior fascicle injuries, indicative of microinstability and lesions of the lateral fibulotalocalcaneal ligament complex (LFTCL: ATFL inferior fascicle + calcaneofibular ligament [CFL]), indicative of macroinstability. Intra‐articular lesions such as soft tissue impingement, osteophytes, loose bodies and osteochondral lesions were also assessed. Preoperative and intra‐operative diagnosis were then compared.

**Results:**

All patients had ATFL injuries. Isolated ATFL's superior fascicle injuries were the most frequent (73.2%), while combined ATFL's superior fascicle and LFTCL injuries accounted for 26.8%. Within LFTCL injuries, ATFL's inferior fascicle was affected in isolation in 21.2% and combined with the CFL in 5.6%. Concomitant intra‐articular lesions were identified in 54.7%, most commonly soft tissue impingement (43.6%), followed by osteochondral lesions (15.1%). Concordance between preoperative and intra‐operative diagnosis was poor (*κ* = 0.08, absolute agreement 24.2%).

**Conclusion:**

Isolated injury of ATFL's superior fascicle is the most frequently observed ligament lesion during arthroscopic treatment of CLAI. Associated intra‐articular pathologies are present in 54.7% of cases. The limited concordance between preoperative and intra‐operative diagnoses in this surgically indicated cohort underscores the role of arthroscopy in the accurate identification and management of intra‐articular lesions.

**Level of Evidence:**

Level III, retrospective comparative study.

**Abbreviations**
ATFL: anterior talo‐fibular ligament
CFL: calcaneofibular ligament
CLAI: chronic lateral ankle instability
LFTCL: lateral fibulotalocalcaneal ligament complex
OL: osteochondral lesion

## INTRODUCTION

Ankle sprain is the most common traumatic condition, accounting for 25% of musculoskeletal admissions to emergency departments [19, 47]. Following an initial episode, 30%–70% of patients report persistent symptoms such as pain or chronic lateral ankle instability (CLAI) [36]. In the long term, CLAI has been identified as the leading cause of post‐traumatic ankle osteoarthritis [15, 27].

These symptoms can be attributed to intra‐articular lesions secondary to the initial trauma, mechanical instability, proprioceptive deficits or weakness of the peroneal muscles [17]. For decades, ankle management focused on physical rehabilitation and non‐operative treatment, with surgery considered a treatment of last choice reserved for severe ligament injuries [29]. Studies have reported intra‐articular injuries in up to 90% of patients with CLAI, leading to the conclusion that the longer the time elapsed since the initial injury, the more severe the associated lesions become [18, 31, 34].

Nowadays, the management of ankle instability has significantly evolved in recent years, with a growing trend toward surgical intervention at less severe stages of ligament injury. This shift is largely driven by a deeper understanding of the anatomy of the ankle ligaments, the pathophysiology of instability and the development of less invasive techniques [37, 45]. Improved recognition and diagnosis of subtle instability, such as microinstability, have enabled clinicians to address instability in its less advanced stages. The findings that anterior talo‐fibular ligament (ATFL) superior fascicle is an intra‐articular but extrasynovial structure with an apparent limited capacity of healing have helped to understand how early treatment can potentially prevent a domino effect in which more ligament damage and additional intra‐articular lesions can appear due to the untreated ankle microinstability [9, 12, 37, 38, 44]. The identification of a wide spectrum of instability patterns, including microinstability or multiligamentous injury such as rotational and dynamic medial instability, has encouraged the adoption of a tailored surgical approach, which is resulting in better clinical outcomes [1, 7, 8, 43]. Finally, the favourable outcomes and shorter recovery times associated with minimally invasive procedures have further supported this transition [16].

However, and despite advances in the management of ankle instability, there is a lack of objective data on incidence of intra‐articular injuries when treatment is performed at less severe stages of ligament damage. Furthermore, few studies have evaluated the specific contribution of arthroscopy in enhancing the accuracy of injury assessment.

Therefore, the primary aim of this study was to describe the arthroscopic findings in terms of ligament injury and intra‐articular pathology incidence in patients operated on a CLAI, with the goal of providing updated insights into the potential benefits of management strategies initiated at less severe stages of ligament injury. The secondary objective was to assess the agreement between preoperative and intra‐operative diagnoses in a cohort of patients undergoing surgery for CLAI. The hypothesis of this study is that current indications for arthroscopic treatment of CLAI increasingly include cases with milder degrees of mechanical instability, which are associated with a lower incidence of intra‐articular pathology compared to the data previously reported.

## METHODS

All patients who underwent arthroscopic treatment for CLAI between 2020 and 2024 were included.

Inclusion criteria were skeletally mature patients with a history of one or more ankle inversion injuries and persistent ankle instability for at least 6 months. Surgical intervention was indicated after failure of a minimum 3‐month course of conservative treatment, which included physiotherapy, rest and anti‐inflammatory medication. Exclusion criteria included any prior foot or ankle surgery, hindfoot malalignment or advanced ankle osteoarthritis. Additional exclusions were the presence of deltoid ligament or syndesmotic injuries identified during arthroscopic exploration, open physes, generalized ligamentous laxity or neuromuscular disorders. Cases lacking complete preoperative clinical data or a full arthroscopic video recording were also excluded from the analysis.

This retrospective study was conducted in accordance with the principles of the Declaration of Helsinki and received ethical approval from our institutional review board (IRB number 2021‐09‐020).

### Preoperative diagnosis

All patients eligible for CLAI surgery underwent a preoperative diagnostic assessment. The preoperative diagnosis was based on patient history and a standardized clinical examination protocol performed by a single examiner, including gait assessment, inspection of ankle appearance, ligament laxity testing and identification of tender points. The following ligaments were evaluated: ATFL and calcaneofibular ligament (CFL). A markedly positive anterior drawer test compared to the contralateral side was considered indicative of an ATFL injury, a positive varus talar tilt test suggested a CFL injury and a positive external rotation test suggested a deltoid ligament injury [1, 35]. A subtle positive anterior drawer test or a posterior drawer test with the ankle in plantarflexion and foot in slight internal rotation, associated with subjective instability and/or anterolateral pain, was suggestive of an isolated injury to the superior fascicle of the ATFL [45].

Intra‐articular pathologies were categorized as soft tissue impingement, presence of osteophytes, loose bodies, ossicles and osteochondral lesions (OLs). Clinically, soft tissue impingement was suspected in cases of tenderness on palpation of the anterolateral or anteromedial aspect of the ankle joint. Osteophytes were suspected in cases of tenderness on palpation of bony prominences or a history of pain during physical activity [26]. Loose bodies were suspected based on a history of joint locking sensations, sudden sharp pain during activity or mechanical clicking. OL was suspected in cases of deep ankle joint pain related to activity and reproducible on palpation [14].

All magnetic resonance imaging (MRI) examinations were independently reviewed by a single examiner to assess the integrity of the lateral ligament complex. The ATFL (superior and inferior fascicle) and CFL were evaluated on axial and coronal planes using proton density–weighted sequences with fat suppression. Ligament status was categorized as intact, partially torn or completely disrupted based on fibre continuity, ligament thickening or thinning, abnormal signal intensity and loss of the normal low‐signal band. Associated indirect signs of injury, including periligamentous oedema, were also recorded [2]. Anterior ankle osteophytes were assessed on sagittal and axial T1 sequences, where osseous spurs are best visualized as low‐signal cortical projections arising from the tibial plafond or talar neck. Soft‐tissue impingement was assessed on fat‐suppressed sequences and considered present when capsular thickening or fibrotic tissue was identified within the anterior tibiotalar recess [4]. OLs of the talus were evaluated on sagittal and coronal T2 sequences. Lesions were defined by the presence of subchondral bone marrow oedema, cartilage surface discontinuity or a clearly demarcated osteochondral defect [24].

Based on the combination of clinical findings and MRI results, ligament injuries were categorized as (a) ATFL superior fascicle injury (referred to as microinstability in the context of this study) or (b) ATFL's superior fascicle associated with a lateral fibulotalocalcaneal ligament complex (LFTCL) injury (ATFL inferior fascicle and/or CFL injury) (referred to as macroinstability in the context of this study) [1, 7, 11, 41, 44]. The overall preoperative diagnosis was based on the type of ligamentous injury and the associated intra‐articular pathologies.

### Surgical technique and intra‐operative diagnosis

All procedures were carried out by a single surgeon specialized in foot and ankle arthroscopy. Arthroscopy was performed in a systematic manner and fully video recorded as part of the patient's permanent record. Patients were placed in the supine position under spinal anaesthesia, with a tight tourniquet. A non‐distraction and dorsiflexion arthroscopic technique was used. The arthroscopic instruments included a 4.0 mm 30° arthroscope, a 3.5 mm arthroscopic shaver and standard arthroscopic instruments. No arthroscopic pump was used.

Cutaneous landmarks over the anterior and lateral aspects of the ankle were highlighted. The standard anterolateral and anteromedial portals and an accessory lateral portal were created. Intra‐articular fatty tissue that generally occupies the central and medial aspect of the anterior ankle compartment was carefully resected with a shaver to allow for complete arthroscopic visualization of the anterior ankle compartment and gutters.

A standardized arthroscopic exploration of the anterior ankle compartment following the seven points previously described [12, 41] was performed for each ankle to ensure that no intra‐articular pathology was missed. This included (1) anterior tibiofibulair ligament's distal fascicle, (2) ATFL's superior fascicle (lateral gutter), (3) lateral talar neck, (4) medial talar neck, (5) deep layer of deltoid ligament and tip of the medial malleolus (medial gutter), (6) medial tibial angle (notch of Henry) and (7) anterior tibial rim. Arthroscopic assessment of the lateral and medial gutters was performed in a slightly dorsiflexed position without distraction, allowing full visualization of the gutters and evaluation of intra‐articular ligaments [5, 20]. Inspection of the talar dome was performed with the ankle in plantar flexion to identify any Grade III–IV OL. The LFTCL was examined with the arthroscope introduced through the anteromedial portal and the probe inserted through the anterolateral portal. Visualization of the fibular tip was considered indicative of a detachment of the ATFL inferior fascicle. Injury to the CFL was suspected when the peroneal tendons were visualized posterior to the fibula. The central and posterior compartments were not evaluated.

**Figure 1:**
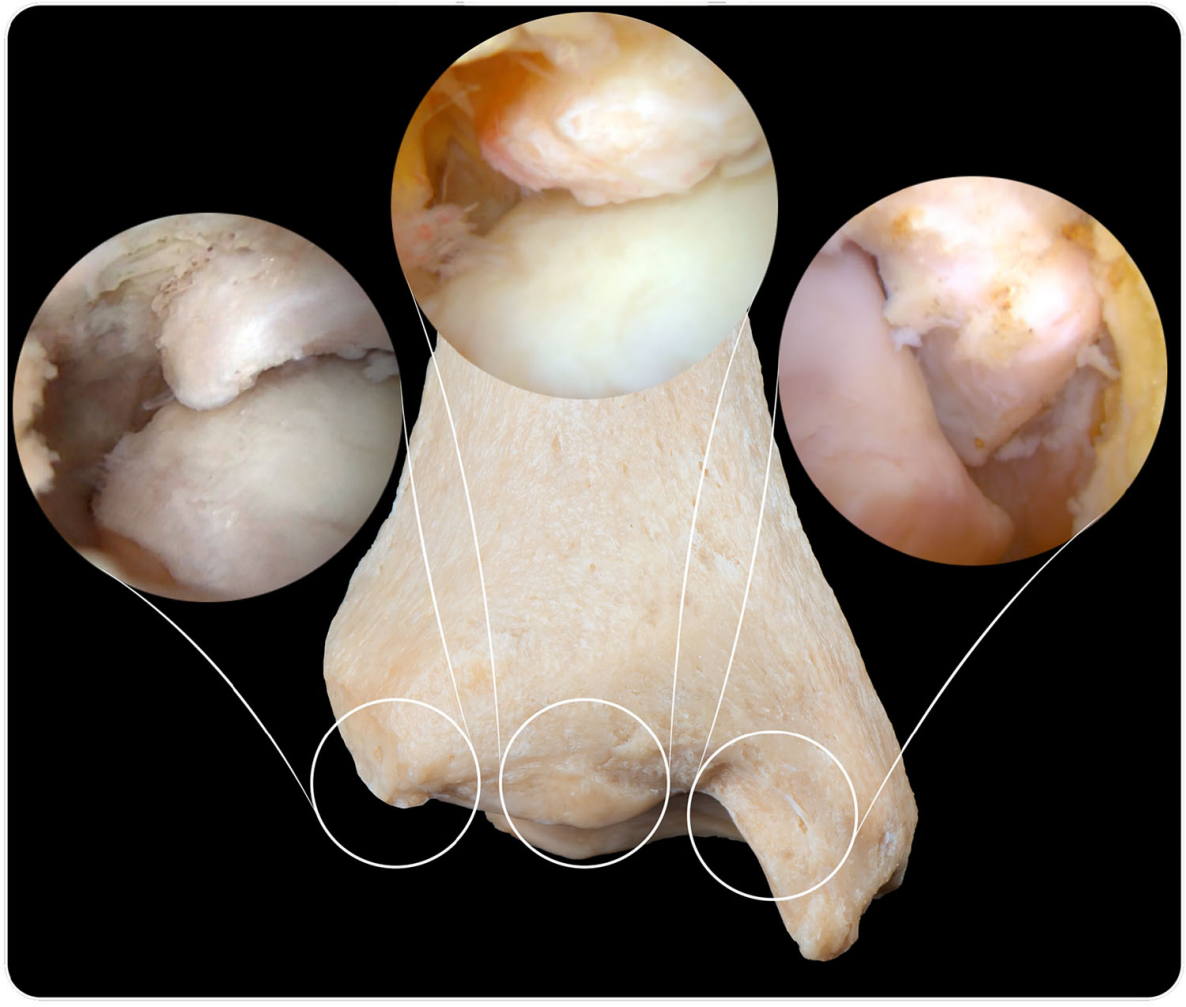
Illustration of tibial zones affected by osteophytes, as identified during arthroscopic exploration. Arthroscopic images demonstrate lateral (Tillaux tuberosity), central (anterior rim) and medial (medial malleolus) osteophytes. Lateral and central osteophytes are visualized through the anteromedial portal, while medial osteophytes are visualized through the anterolateral portal.

**Figure 2:**
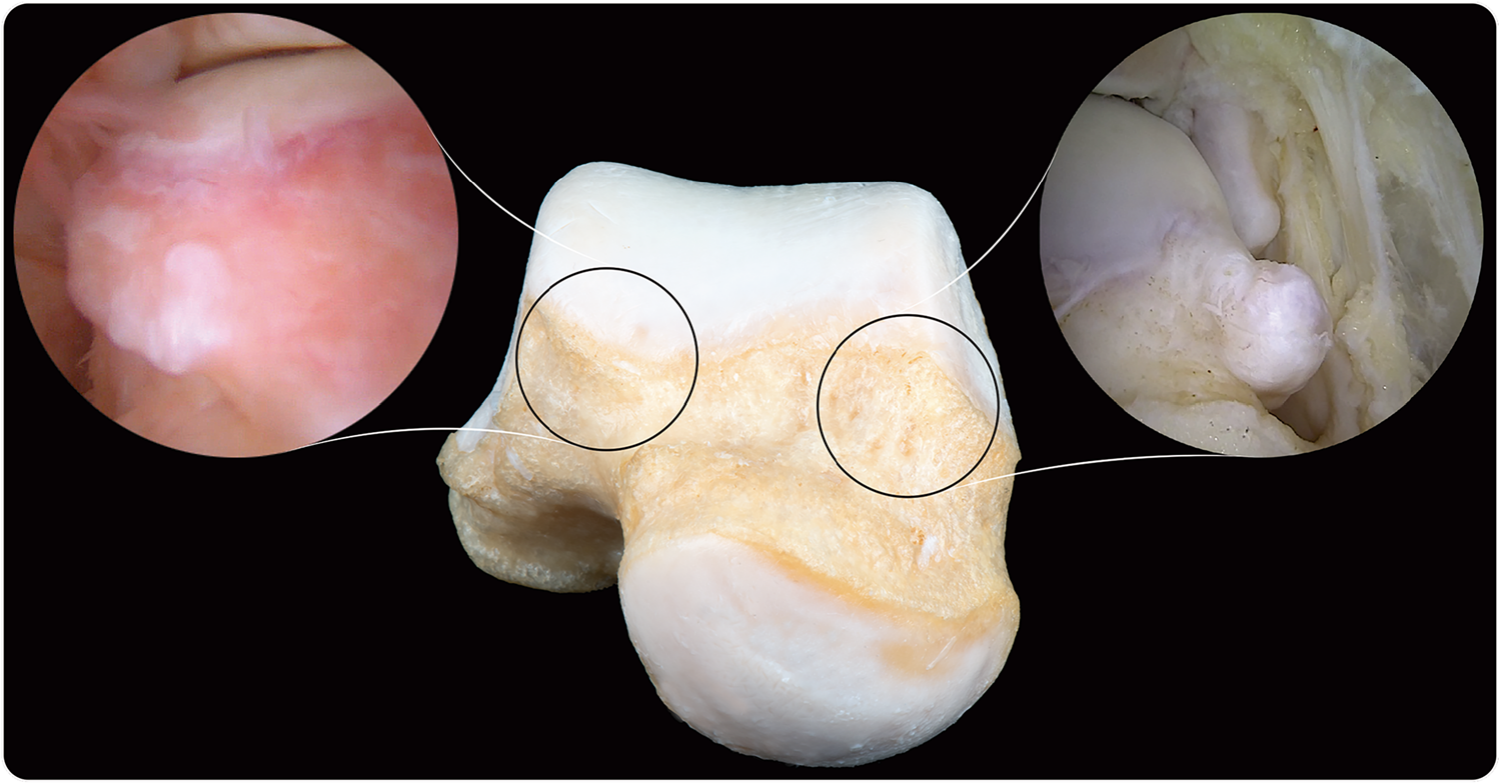
Illustration of talar zones affected by osteophytes identified during arthroscopic exploration. Arthroscopic images depict lateral and medial talar osteophytes.

The presence of any intra‐articular pathology and type of ligament injury observed during arthroscopic examination was noted and recorded in a table by one of the coauthors. Intra‐articular pathology included soft tissue impingement (synovitis or web impingement), OL (on the talus or on the tibia), intra‐articular loose bodies, ossicles and bony osteophytes. OL were assessed by inspection and probing and were located using the schematic nine‐zone grid of the talar dome described by Raikin et al. [30]. Tibial osteophytes were divided into medial (medial malleolus), central (anterior rim) and lateral (Tillaux tuberosity) (Figure 1). Talar osteophytes were divided into medial and lateral (Figure 2). If needed, pathological soft‐tissue debridement with arthroscopic motorized shaver was carefully performed to preserve the ligamentous structure and to observe the anatomy of the lateral and medial collateral ligaments.

Arthroscopic recordings were reviewed by one of the co‐authors not involved in the surgery and double checked with the intra‐operative records to be sure to not lose any intra‐articular pathology. Discrepancies between the operator and the independent reviewer were resolved by joint reassessment of the recordings until consensus was achieved. For each case, an intra‐operative diagnosis was established on the base of ligament injury and intra‐articular pathology observed during arthroscopic exploration. Ligamentous and intra‐articular injuries were arthroscopically treated accordingly. In cases of ligament injury, they were repaired using the all‐inside technique as previously described [17, 40, 42].

### Statistical analysis

Statistical analyses were conducted using SPSS Statistics© software (v.28, IBM©).

Descriptive statistics were used to analyse the frequency of intra‐articular findings observed during arthroscopy. Categorical data were expressed as percentages, including the presence of osteophytes, bony impingement, soft tissue impingement, OL and loose bodies. A McNemar test was used to compare pre‐ and intra‐operative findings.

Agreement between preoperative and intra‐operative diagnoses has been assessed using Cohen's *κ* coefficient. Interpretation of the *κ* value followed Landis and Koch's criteria: values < 0.20 indicate slight agreement, 0.21–0.40 fair, 0.41–0.60 moderate, 0.61–0.80 substantial and >0.80 almost perfect agreement.

## RESULTS

During the study period, 229 patients underwent arthroscopic treatment for CLAI. A total of 50 patients were excluded, 44 had a deltoid ligament injury, 4 had a history of prior ligament surgery on the same ankle, and 2 had constitutional ligamentous laxity. A total of 179 patients were included in the analysis. The mean age was 30.4 ± 14.5 years (range from 16 to 77). There were 104 males (58.1%) and 75 females (41.9%). The right ankle was affected in 90 cases (50.3%) and the left ankle in 89 cases (49.7%).

### Intra‐operative findings

The intra‐operative findings are detailed in Table 1.

**Table 1:** Findings of ligaments and intra‐articular lesions based on arthroscopic evaluation.

Pathology identified	Intra‐operative diagnosis
	*n* (%)
Ligament injury	
Isolated ATFL superior fascicle	131 (73.2)
ATFL SF + LFTCL	48 (26.8)
ATFL inferior fascicle	38 (21.2)
ATFL IF + CFL	10 (5.6)
Intra‐articular injuries	
Soft tissue impingement	78 (43.6)
Web impingement	16 (8.9)
Synovitis	62 (34.6)
Osteophytes	28 (15.6)
Loose bodies	8 (4.5)
Fibular ossicles	2 (1.1)
Osteochondral lesions of talus	24 (13.4)
Osteochondral lesions of tibia	2 (1.1)
Kissing lesion	1 (0.6)

Abbreviations: ATFL IF, anterior talo‐fibular ligament inferior fascicle; ATFL SF, anterior talo‐fibular ligament superior fascicle; CFL, calcaneo fibular ligament; LFTCL, lateral fibulotalocalcaneal ligament complex.

The most frequent ligament injury identified intra‐operatively was isolated detachment of ATFL's superior fascicle, identified in 131 cases (73.2%). In the remaining 48 cases (26.8%), an associated injury of the LFTCL complex was observed. Of these, in 38 cases (21.2%), the injury affected only the ATFL component (inferior fascicle) of the LFTCL complex, while in 10 cases (5.6%) the injury affected both the ATFL and the CFL component of the LFTCL complex. Then, intra‐operative diagnosis of microinstability was established for 131 patients (73.2%), while macroinstability was diagnosed in 48 patients (26.8%).

Arthroscopic exploration identified intra‐articular pathologies in 98 patients (54.7%). The most frequent finding was soft tissue impingement, observed in 78 patients (43.6%), followed by osteophytes in 28 cases (15.6%). Osteophyte's location is detailed in Table 2. OLs were found in 27 cases (15.1%). Among them, 24 were located on the talus (13.4%), 2 were located on the tibia (1.1%), and 1 combined OL of talus and tibia (kissing lesion) was found in one case (0.6%). Fibular ossicles were found in 2 cases (1.1%) and loose bodies were identified in 8 cases (4.5%).

**Table 2:** Details of osteophytes' location based on arthroscopic evaluation.

Osteophyte location	*n* (%)
Tibial	14 (7.8)
Medial osteophytes	6 (3.4)
Lateral osteophytes	2 (1.1)
Central osteophytes	10 (5.6)
Talus	16 (8.9)
Medial osteophytes	12 (6.7)
Lateral osteophytes	3 (1.7)
Central osteophytes	2 (1.1)

**Figure 3:**
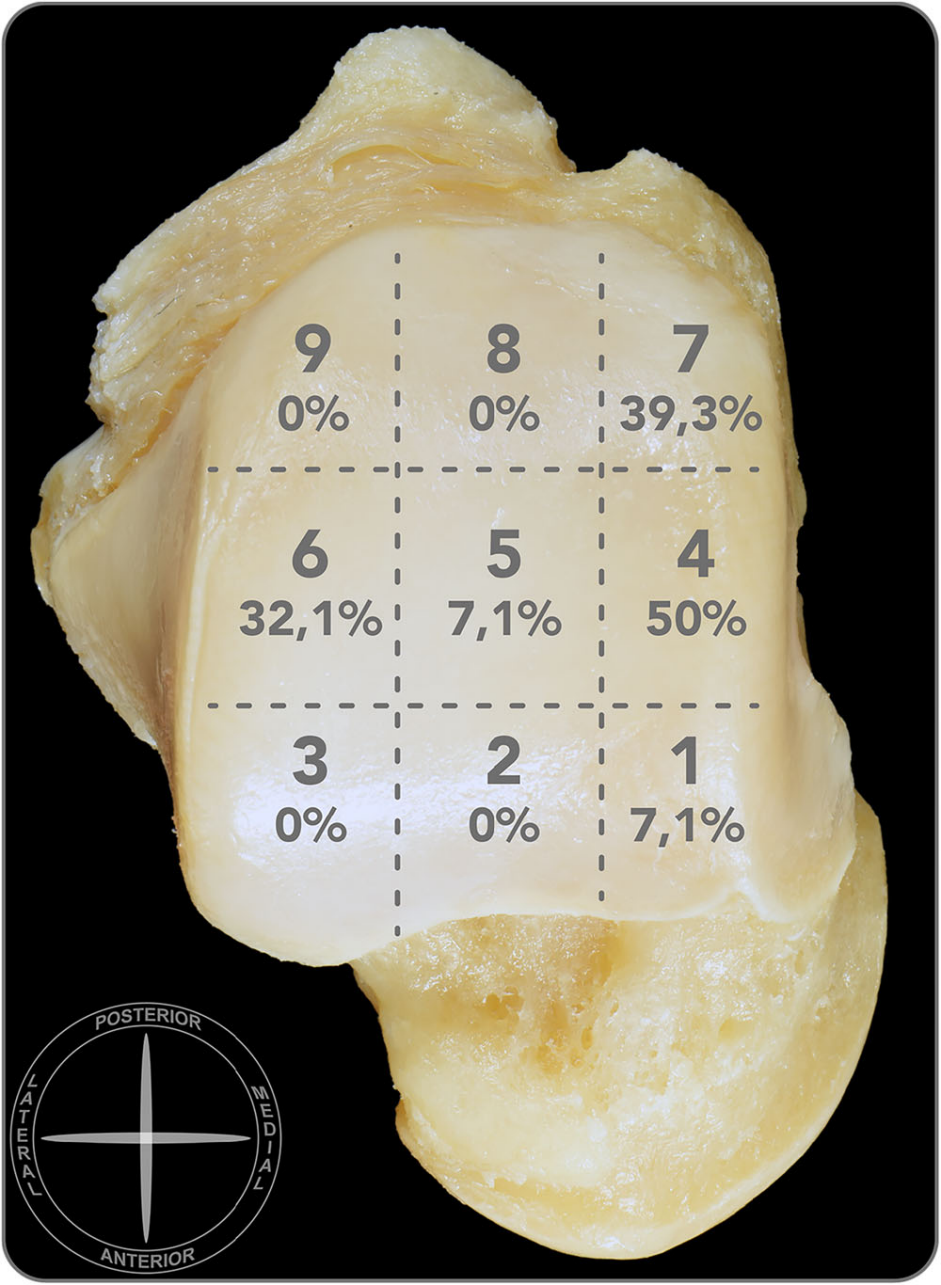
Distribution of osteochondral lesions across talar dome zones according to Raikin classification, as observed during arthroscopic evaluation.

OL of talus most involved the medial third of the talar dome (64.2%, *n* = 18), followed by the lateral third (32.1%, *n* = 9) and central third (7.1%, *n* = 2). In one case, the OL involved both medial and central third. A total of eight OL of the talus involved more than one zone. Repartition of the zones of the talar dome affected by OLs according to Raikin's classification is detailed in Figure 3. Patients with LFTCL involvement demonstrated a higher rate of postoperative OLT compared with isolated ATFL's superior fascicle lesions (19.6% vs. 10.7%); however, this difference did not reach statistical significance (OR 0.49, 95% CI 0.20–1.23; *p* = 0.13).

### Comparison between preoperative and intra‐operative diagnosis

Preoperative findings were significantly different in comparison with intra‐operative findings except when a diagnosis of combined lesions of ATFL and CFL was made (Table 3).

**Table 3:** Comparison between expected (preoperative diagnosis) and arthroscopically confirmed ligament and intra‐articular findings.

Pathology identified	Preoperative diagnosis	Intra‐operative diagnosis	Difference	
	*n* (%)	*n* (%)	*n* (%)	*p* value
Ligament injury				
Isolated ATFL superior fascicle	96 (53.6)	131 (73.2)	35 (15.7)	<0.001
ATFL SF + LFTCL	82 (45.8)	48 (26.8)	34 (19)	<0.001
ATFL inferior fascicle	75 (41.9)	38 (21.2)	37 (20.7)	<0.001
ATFL IF + CFL	7 (3.9)	10 (5.6)	3 (1.3)	*p* = 0.248
Intra‐articular injuries				
Soft tissue impingement	2 (1.1)	78 (43.6)	76 (34.1)	<0.001
Tibial osteophytes	5 (2.8)	14 (7.8)	9 (4)	<0.05
Talar osteophytes	7 (3.9)	16 (8.9)	9 (4)	<0.05
Loose bodies	2 (1.1)	8 (4.5)	6 (2.7)	<0.05
Fibular ossicles	2 (1.1)	2 (1.1)	0 (0)	1
Osteochondral lesions	16 (8.9)	27 (15.1)	8 (3.6)	<0.05

Abbreviations: ATFL IF, anterior talo‐fibular ligament inferior fascicle; ATFL SF, anterior talo‐fibular ligament superior fascicle; CFL, calcaneo fibular ligament; LFTCL, lateral fibulotalocalcaneal ligament complex.

Preoperative and intra‐operative overall diagnoses were identical in 24.2% of cases. The Cohen's *κ* coefficient between preoperative and intra‐operative diagnoses was 0.08, indicating a slight agreement. Considering only the ligament diagnosis, preoperative and intra‐operative diagnoses were identical in 41.9%. The Cohen's *κ* coefficient between preoperative and intra‐operative diagnoses was 0.20, indicating a slight agreement. Considering only the intra‐articular injuries diagnosis, preoperative and intra‐operative diagnoses were identical in 43.6%. The Cohen's *κ* coefficient between preoperative and intra‐operative diagnoses was 0.09, indicating a slight agreement.

## DISCUSSION

In this retrospective study of 179 patients who underwent arthroscopic surgery for CLAI, our main finding was the predominance of isolated injuries to the superior fascicle of the ATFL, an injury consistent with the diagnosis of microinstability. While 54.7% of patients presented with concomitant intra‐articular lesions, this rate appears to be lower than that reported in previous studies. Most of these lesions were soft tissue impingements (43.6%), OLs of the talus were identified in 13.4% of patients. These findings suggest that treatment of ankle instability at less severe stages of ligament injury may help reduce the risk of intra‐articular damage. Additionally, preoperative diagnostic assessments demonstrated poor concordance (24.2%) with intra‐operative findings, highlighting the added value of arthroscopy in accurately detecting intra‐articular injuries.

The most common ligament injury identified in our study was an isolated lesion of the superior fascicle of the ATFL observed in 73.2% of cases. Superior ATFL fascicle is the first ligament damaged after a lateral ankle sprain because of its stabilizing role in plantar flexion [10]. Its intra‐articular but extrasynovial location is expected to compromise its healing, most often resulting in a mild mechanical instability, referred to in the present study as microinstability based on previous anatomical and arthroscopic descriptions [10]. The rate of microinstability reported in our study is similar to that described by Vega et al. in 2014 [46]. Unrecognized microinstability may initiate a cascade of events, leading to recurrent ankle sprains and progressive mechanical instability. This, in turn, can contribute to the development of more severe injury patterns, including LFTCL involvement (macroinstability), deltoid ligament compromise, articular cartilage degeneration and osteophyte formation associated with rotational instability [3, 9, 28, 40].

In our series, LTFCL was involved in 26.8% of cases, including 21.2% of ATFL inferior fascicle injuries and 5.6% of combined ATFL and CFL injuries, which appears lower than the rates reported by Hintermann and Schaffer, who described 64% and 69% of combined ATFL and CFL injuries, respectively [5, 18]. This may reflect significant advances in the management of ankle instability over the past two decades, along with increased awareness of the concept of microinstability, which has prompted surgical intervention at less severe stages of ligament injury and may help prevent the progression of ligament damage [23, 25, 39, 46].

Similarly, the rate of intra‐articular injuries observed in our study (54.7%) lies toward the lower range of those reported in the literature, including the series of Hua et al. published in 2010, which reported a rate of 90% [21]. Among these lesions, 13.4% of our patients presented OL of the talus, a proportion that also appears lower than previously reported rates of 54% and 55% by Schaffer et al. and Hintermann et al., respectively [5, 18]. To the authors' knowledge, recent data specifically addressing the prevalence of intra‐articular lesions in arthroscopically treated CLAI remain limited.

Osteochondral damage has been shown to alter joint biomechanics and may progress to osteoarthritis [6, 32]. In this context, early management of ankle instability may be of interest to limit the progression of intra‐articular damage. Although statistical significance was not reached, a higher prevalence of postoperative OLs was observed in patients with severe ligamentous injury (LTFCL) compared with isolated ATFL superior fascicle injuries (19.6% vs. 10.7%). These findings suggest a potential association between ligament injury severity and osteochondral damage, which may have been underpowered in the present cohort. Larger studies are warranted to further investigate this relationship.

The rates of soft tissue impingement and osteophytes in our study (43.6% and 15.6%, respectively) were consistent with those previously reported in the literature. Such impingements can cause various symptoms, including pain and limited range of motion, and likely contribute to the high rate of persistent symptoms following lateral ankle sprains [33]. Arthroscopic treatment of soft tissue impingement has already demonstrated its effectiveness [22, 33]. Addressing ankle instability at earlier stages may also allow concomitant management of frequently associated intra‐articular lesions, including soft tissue impingement, although the impact on long‐term outcomes remains to be determined.

The agreement between preoperative and postoperative diagnoses remains poor. Although MRIs have demonstrated the ability to distinguish between the superior and inferior fascicles of the ATFL, identifying an isolated lesion of the superior fascicle remains challenging, making the diagnosis of microinstability difficult [13, 20]. The greatest discrepancy between preoperative and postoperative diagnoses was observed in cases of soft tissue impingement, reflecting the limited diagnostic accuracy of MRI for this condition [48]. In this context, arthroscopy remains the only tool that enables comprehensive exploration, accurate diagnosis and simultaneous treatment of intra‐articular ankle pathology.

Our study has several limitations. Its retrospective design introduces potential selection and information bias, as data collection relied on existing records and operative documentation. Also, all procedures and pre‐operative assessments were conducted by a single experienced surgeon, which, while ensuring consistency, may limit the generalizability of the findings to other clinical settings. A multicentric study could have enhanced the external validity of the study. A further limitation of this study is that only high‐grade OL (Grades II–IV) were visualized arthroscopically, whereas lower‐grade lesions (Grades I–II), which are likely more prevalent, were not assessed, potentially underestimating the overall incidence of OL. Lastly, although a standardized arthroscopic protocol was applied, the assessment of ligament integrity and intra‐articular pathology remains partly subjective, potentially introducing variability in the interpretation of subtle lesions. To mitigate this limitation, the arthroscopic records were reviewed independently by a co‐author. However, a formal evaluation by two fully independent reviewers would have further strengthened the reliability of the assessments.

## CONCLUSION

Isolated injury of ATFL's superior fascicle, indicative of microinstability, is the most frequently observed ligament injury during arthroscopic treatment of CLAI. Associated intra‐articular pathologies are present in 54.7% of ankles with chronic instability. In addition, the limited concordance between preoperative diagnosis and intra‐operative diagnosis in this cohort of patients undergoing surgery for CLAI highlights the role of arthroscopy in the accurate identification and management of intra‐articular lesions.

## AUTHOR CONTRIBUTIONS

**Pierre‐Henri Vermorel**: Statistical analysis, manuscript drafting. **Jordi Vega**: Study conception and design, critical manuscript revision. **Miki Dalmau‐Pastor**: Illustrations, manuscript revision. **Andrea Pantalone**: Data collection, statistical analysis. **Javier Zaourak**: Manuscript modification, conception and design. **Matteo Guelfi**: Data collection, study conception and design, critical manuscript revision.

## CONFLICT OF INTEREST STATEMENT

The authors declare no conflict of interest.

## ETHICS STATEMENT

This study was conducted in accordance with the principles of the Declaration of Helsinki and received approval from our Institutional Review Board (IRB number 2021‐09‐020).

## Data Availability

The authors have nothing to report.
